# Protocol for an exploratory, longitudinal single case study of a novel palliative care rehabilitative service

**DOI:** 10.12688/hrbopenres.13461.2

**Published:** 2022-05-06

**Authors:** Fódhla N. Ní Chéileachair, Bridget M. Johnston, Cathy Payne, Fiona Cahill, Lisa Mannion, Lisa McGirr, Karen Ryan

**Affiliations:** 1 Department of Palliative Care, Mater Misericordiae University Hospital, Dublin, Dublin, Ireland; 2 Centre for Health and Policy Management, Trinity College Dublin, Dublin, Ireland; 3 All Ireland Institute of Hospice and Palliative Care, Dublin, Ireland; 4 St. Francis Hospice, Dublin, Ireland

**Keywords:** Palliative Care, Rehabilitation, Study protocol, Quality of Life

## Abstract

**Background: **Early access to rehabilitation can improve quality of life for those with life-limiting illnesses and is highlighted as a core component of the Adult Palliative Care Services Model of Care for Ireland. Despite this, palliative rehabilitation remains under-utilised and under-developed. In 2020, the Sláintecare Integration Fund provided opportunity to pilot a novel rehabilitative palliative care service, “Palliat Rehab”. This protocol proposes a case study, which aims to advance understanding of the form, content, and delivery of the pilot service.

**Methods: **A prospective, longitudinal, mixed-methods, case study design will be used to describe the service and to explore the experiences of patients, informal carers and clinicians. Additionally, data collection instruments will be tested and the utility of outcome measures will be examined. Data will be collected from documentary, survey, and interview sources.

Quantitative data will be analysed using descriptive statistics, including chi-square tests for categorical variables, Mann-Whitney U tests for ordinal data, and t-tests/ ANOVA for continuous data. Qualitative data will be analysed using thematic analysis.

**Conclusions: **New pathways are required to advance service provision to ensure that patients receive the ‘right care, in the right place, at the right time’. This protocol outlines a case study which will aim to develop current understanding of the implementation and delivery of a novel rehabilitative palliative care service in Ireland and will consider its potential contribution to the achievement of Sláintecare goals. Investigating the service within its environmental context will lead to a better understanding of ‘how’ and ‘why’ things happen. Findings will be used to inform efforts to further develop and tailor the intervention.

## Introduction

Rehabilitative palliative care has been defined as a paradigm integrating rehabilitation, enablement, self-management, and self-care into the model of palliative care to provide patients with support to enjoy the fullest possible life until death ^3^. The unmet rehabilitative needs of those with serious illness are increasingly recognised- that is, the need to be normal and in control, the need for better every day functioning and mobility, and the need to alleviate the fear of being a burden ^4^

Interventions adopting a rehabilitative palliative care approach can benefit individuals with life-limiting illnesses ^48– 52^. Specific studies have pointed to the benefit of an exercise programme for patients with cancer-related fatigue ^6^ and advanced non-small cell lung cancer ^7^. Recently, Nottelmann and colleagues conducted a randomised controlled trial of a novel, integrated rehabilitative palliative care programme for individuals newly diagnosed with advanced cancer and identified a benefit to their quality of life from the programme ^8^. These results are increasingly relevant in the Irish context, as the number of older people living with a palliative care need in Ireland is projected to increase by 89% between 2016 to 2046 ^1^. This population will account disproportionately for disability burden, pain prevalence and health care use, leading to increased population health burdens and health care utilisation. It is estimated, for example, that there will be a 106% increase in requirement for the hours of healthcare delivered at home ^1^. As such, rehabilitative care offers an important way for the Irish health system to re-tool to meet these challenges, yet it is an under-developed component of palliative care for all adults receiving services in Ireland ^2^.

Though there may be substantial benefits to be gleaned from adopting a rehabilitative palliative care approach, difficulties implementing the approach have been noted internationally. In 2016, Thuesen and colleagues ^5^ conducted a literature review on the international evidence for coordination of rehabilitation and palliative care. This extensive review made notable recommendations for the implementation of rehabilitation in the palliative care approach, such as a coordinated programme that should be individualised to the needs of the patient, dynamically revised to suit their progress, and effectively assessed using outcome measures for all programme elements. Despite the growing evidence base for the benefits of offering rehabilitation programmes for individuals with palliative care ^48– 52^, the publication of Thuesen and colleagues ^5^ also highlighted substantial gaps in available guidelines for coordinating palliative care and rehabilitation in a single care approach. The authors concluded that there remained a lack of consensus on when and how rehabilitative palliative care should be offered and recommended that further research was required with regards to methods, activities, frameworks, and practical guidelines for service provision ^5^. As such, the current knowledge base on designing, coordinating, and delivering rehabilitative palliative care interventions remains incomplete and requires greater development to facilitate the coordination of these approaches.

### Rehabilitative Palliative Care in Ireland 

In Ireland, the few studies which have been conducted to date on palliative care rehabilitation have been descriptive or exploratory in nature ^9– 11^, and the only interventional study that has been published has focused on health and social care professional education ^12^. Rehabilitation was, however, highlighted in the Adult Palliative Care Services Model of Care for Ireland produced in 2019 ^2^. The Adult Palliative Care Services Model of Care for Ireland recognises the importance of rehabilitative palliative care as a core part of service provision, while acknowledging the deficits that exist in staffing and development of services ^2^. The aim of the model of care is to ensure all individuals with a life-limiting illness in Ireland have access to the appropriate level of palliative care services to enhance their quality of life, irrespective of diagnosis and care setting. Included in the model of care is the optimal pathway of care for individuals with life-limiting illnesses, which entails a thorough needs assessment, initiating the correct care (e.g., specialist, generalist, integrated services), planning for ongoing and future care needs, engaging end-of-life care supports, and care for loved ones through the process of bereavement. Overall, the model of care acts as a guiding document for palliative care provision using eight core foundations to deliver an accessible and appropriate palliative care service.

Given the increasing demand for palliative care services in Ireland and the notable benefits of a rehabilitation approach in palliative care, greater coordination between palliative care and rehabilitation services is likely to prove a valuable approach in the health service. Greater integration of rehabilitation into palliative care in Ireland also aligns with the goals of healthcare reform in Ireland, as outlined in the Sláintecare Report. Established in 2017, Sláintecare is a ten-year reform programme in Ireland aimed at providing a more efficient and universal healthcare services, including the commitment to develop universal palliative care ^13, 53^. Responding to the urgent need to provide support to the healthcare system to test and scale ideas that meet Sláintecare goals, the Sláintecare Integration Fund was established in 2019. The Fund aims to support projects that:

- Demonstrate innovative ways in which citizens can engage in their own health,
- Represent best practice in the management of chronic diseases and caring for older people, and
- Encourage innovations in shift of care to the community or promoting hospital avoidance.

Following application to the Fund, our team was awarded a grant to deliver an integrated rehabilitation service for patients with palliative care needs and to conduct an exploratory, longitudinal case study. The novel intervention draws on the core foundations of the Model of Care ^2^ as a means of enhancing research and innovation capacities in palliative care by examining the feasibility of providing the integrated service between hospital and community care settings. A description of each core foundation is available in the *Extended Data,* accompanied by a brief outline of elements in the intervention drawing upon each one. The case study aims to advance understanding of the form, content, and delivery of the novel rehabilitative palliative care service (‘‘Palliat Rehab’’) in the provision of specialist palliative care for academic, policy and practice purposes.

### Objectives

This is a protocol for a case study which will examine a palliative rehabilitation intervention. The specific study objectives are:

- To describe the novel rehabilitative palliative care service that spans hospital and community settings.
- To explore the experiences of patients, informal carers and clinicians while receiving or providing the service.
- To test data collection instruments and examine the utility of selected outcome measures.

## Methods

### Design

Guided by an exploratory case study framework ^16^, and adopting a post-positivist approach ^17^, this case study will examine the novel service, as delivered by one hospital organisation and one community-based organisation, for an 18-month period of service provision and will provide insight into the delivery of palliative care rehabilitation. ‘Palliat Rehab’ is a new service initiative, and a case study design was chosen because of the need to capture information to answer ‘how’, ‘what’ and ‘why’ questions using a naturalistic method ^14^. Case studies allow for investigation of a contemporary phenomenon within its real-life context when the boundaries between phenomenon and context are not clearly evident ^14^ and provide an in-depth, multi-faceted understanding of issues that can help develop or refine theory ^15^.

The scope of our study includes the rehabilitation service, the staff delivering the service, and the direct and indirect recipients of the service (patients and informal caregivers/family members). This study will comprise two components of data collection: a ‘concurrent’ component, where routine, quantitative data will be collected from participants during the intervention sessions, and a ‘phased’ component, where patients will be invited to contribute data on their experiences of Palliat Rehab using a quantitative survey and during semi-structured, qualitative interviews. Their informal caregivers and relevant staff members will also be invited to engage in semi-structured interviews on their views of the service as an additional element of the phased component. In doing so, the study will contribute to opening the ‘black box’ of palliative care rehabilitation interventions and provide transferable knowledge that will be of value to the future development of interventional studies of service delivery.

### Study setting

The rehabilitation service will be provided by the Mater Misericordiae University Hospital (Mater Hospital) and St Francis Hospice for an 18-month period.

The Mater Hospital is a 630-bed teaching hospital providing local services for its catchment population of Dublin’s north inner city and a range of specialist services on a regional and national level. St Francis Hospice Dublin is a specialist palliative care service organisation providing in-patient, out-patient, and home-based services to people with progressive, life-limiting illness in the North Dublin area. The rehabilitation service will be delivered by a hospital-based senior occupational therapist and a community-based senior physiotherapist, both of whom work as members of specialist palliative care teams.

### Eligibility Criteria

The case study will recruit participants from the specialist palliative care services of the Mater Hospital and the community palliative care services of St Francis Hospice Dublin. Overall, the following three groups of participants have been identified and will be recruited during this case study: patients who receive Palliat Rehab, informal caregivers/ family members of patients receiving the rehabilitation service, and staff engaging in either the design or delivery of Palliat Rehab.

For patients receiving palliative care in the Mater Hospital or in St. Francis Hospice, referral to Palliat Rehab will depend on a palliative needs assessment, where severe ill-health, symptom burden and/or cognitive deficit may preclude individuals from taking part. Needs assessments comprise holistic assessments of individual need that include, but are not limited to, assessment of physical condition, appraisals of pain, examination of function and mobility, discussion of potential difficulties completing activities of daily living, and identifying goals patients may have for their care. As rehabilitation needs can vary substantially between individuals and their conditions, however, inclusion will be determined on a case-by-case basis. If the specialist palliative team think that an individual has a rehabilitation need that could be met by the service, then the new service will be explained and offered to the patient by a team member. Specific eligibility criteria for each group have been highlighted in Table 1, where criteria for patients refers to both the concurrent and phased components, and criteria for caregivers and staff refers to the phased component. 

**Table 1.** Eligibility criteria for components of the study.

Patient inclusion criteria *Concurrent and phased components*	Caregiver inclusion criteria *Phased component*	Staff inclusion criteria *Phased component*
1. Receiving the novel palliative care rehabilitation service due to the identification of a rehabilitation need 2. ≥ 18 years of age 3. English speaking 4. Able to provide informed consent 5. Patients do not need a caregiver willing to participate in the study	1. Self-endorsing or identified by the patient as a primary caregiver 2. ≥ 18 years of age 3. English speaking 4. Able to provide informed consent 5. Caregivers related to a participating patient are eligible for inclusion if the above criteria are satisfied, regardless whether their loved one participates in the research element or receives the intervention only.	1. Identified as a clinical staff member who has been involved in the design and/or delivery of the palliative care rehabilitation service
Patient exclusion criteria	Caregiver exclusion criteria	
1. Unable to complete the study measures and/ or participate in an interview in the opinion of attending clinician. Patients may be unable to complete measures or participate due to ill-health, symptom burden or cognitive impairment.	1. Patient does not wish caregiver to be invited to participate in the study	

### Intervention

Palliat Rehab will be delivered by an occupational therapist in the Mater Hospital and a physiotherapist in St. Francis Hospice. It has been observed that rehabilitation is best described as a process containing specific actions ^21^, and for this reason, the bundle of interventions, based on individual rehabilitation needs, will be investigated rather than specific components. The occupational therapist and physiotherapist delivering Palliat Rehab will engage with the patients directly about their rehabilitation wants and needs and will construct individualised plans on a case-by-case basis. As the specific elements of each patient’s experience will vary, no set number of intervention sessions have been planned. The number of intervention sessions will depend on the patient’s health, their desire to continue with the service, and their ongoing connection with either the Mater Hospital and/or St. Francis Hospice. A description of the intervention service based on TIDIER criteria is available in the *Extended data.*

### Outcomes

This case study will collect multiple outcome measures to describe the intervention, to explore the experiences of participants, and to assess the utility of particular quantitative measures for such an intervention. The quantitative outcome measures to be collected from consenting patient participants during the concurrent component are divided into three categories (demographic, service-usage, and health and symptom data) and are listed in Table 2. By collecting these three categories of data, it is anticipated that this case study will be equipped to adequately describe the sample served, the experience of patients receiving the intervention, and the feasibility of embedding the service into the existing healthcare infrastructure using service-usage data. All quantitative data measures will be collected once following initial enrolment of each patient, with the exception of the Palliative Care Outcomes Collaborative (PCOC) scale, which will be collected at baseline during the first intervention session and repeatedly collected during each session thereafter.

**Table 2.** Quantitative data and measures.

Demographic data	Service-usage data	Health and symptom data
1. Gender Identity 2. Age 3. Marital status 4. Primary diagnosis 5. Primary care network 6. Current living conditions including home supports	1. Response times between referral and service enrolment 2. The number, type, nd period of clinical interactions with the rehabilitative palliative care service 3. Length of hospital stay (for hospitalised patients only) 4. Whether re-admitted to hospital within 30-days of discharge or receipt of services enrolment 2. The number, type, and period of clinical interactions with the rehabilitative palliative care service 3. Length of hospital stay (for hospitalised patients only) 4. Whether re-admitted to hospital within 30-days of discharge or receipt of services	1. The Palliative Care Outcomes Collaborative (PCOC) scale ^22^ 2. Charlson Comorbidities Index (CCI) ^23^ 3. The Clinical Frail Scale ^24^ 4. Palmar Grip Strength 5. 5x Sit-to-Stand test ^25^

For the phased component, patients receiving Palliat Rehab will be asked if they are willing to contribute survey data based on their satisfaction with the service. Outcomes relating to the phased component of the case study also include the qualitative interview data collected from all three participant groups to effectively explore the general experiences and views of the service from the patient, informal caregiver, and staff perspective. 

### Sample size

The sample size for a case study depends on the number of participants that is sufficient to describe the phenomenon ^18^. As this case study design comprises two components, namely the concurrent component and the phased component, the rationale for each component’s sample size is described below.

### Concurrent Component

The aim of the case study is not to test the rehabilitation service, therefore it would not be appropriate to use traditional power calculations when considering sample size for the quantitative data collection component of the case study. ^20^ The purpose of quantitative data collection in the case study is to contribute to the understanding of care delivery and stakeholder experience and to test data collection instruments and examine the utility of selected outcome measures. Considering the issue of justification of sample adequacy, anonymised, routine data will be collected on all patients who receive the service. , and all eligible patients will be offered the opportunity to participate in survey data collection.

### Phased Component

Following the concurrent component, all patients who receive Palliat Rehab will be offered the opportunity to participate in a survey detailing their satisfaction with the service. The second element of the phased component refers to the qualitative interviews. As sample size estimation for qualitative interview samples is an area of conceptual debate and practical uncertainty ^19^, the projected sample size is based on the concepts of data adequacy and research practicality. The population included within this case study is relatively homogeneous, as participants will be availing of one single palliative care rehabilitation service. A sample of 16–22 individuals will therefore be recruited for qualitative data analysis across three stakeholder groups; patients receiving the service *(n= 4*–6), family members of individuals receiving the service ( *n*= 4–6), and clinicians involved in the design and/or delivery of the new service ( *n*= 8–10).

### Recruitment

***Patients.*** All potential participants will be provided with an information leaflet detailing the purpose of the data collection, the potential risks and benefits to participation, and data protection rights. Following a minimum of 24 hours, potential participants will be asked whether they are interested in being contacted by a researcher to discuss participation in the concurrent and phased components. Patients will be assured that they may receive Palliat Rehab through the concurrent component without further engaging in the phased component of the study. Once a patient receives input from the service, a researcher will make telephone contact with individuals who indicate that they are willing to be contacted and enquire if they are interested in participating in the phased component.

***Caregivers.*** Patients receiving ‘Palliat Rehab’ will also be asked by either the physiotherapist, occupational therapist, or consultant to consider whether they wish for their primary caregiver to also be invited to participate in a separate Zoom or telephone interview. It will be explained that the reason for inviting their caregiver to participate is to include caregiver perspectives in the research study. It will again be emphasised that their decision or that of their caregiver will not influence the patient’s care in any way. Following a minimum of 24 hours and allowing sufficient time for the patient to discuss the matter with his/her caregiver, the patient will be asked whether their caregiver is interested in being contacted by a researcher to discuss participation. A researcher will telephone individuals who indicate that they are willing to be contacted and answer questions and provide information, as needed.

***Staff.*** Clinical staff who have been involved in the design and/or delivery of the palliative care rehabilitation service will be invited to take part in two interviews by the Principal Investigator (PI). The researcher will provide the potential participant with written information on the study and the consent form and will be available to answer further questions, as needed. Both interviews will be separated by a minimum of 6 months to contrast learnings and applications of developed knowledge during the piloting of the intervention. It will be explained that the reason for inviting the staff member to participate is to include the perspectives of health and social care professionals in the study. Information will be provided to them regarding the study, the potential risks and benefits to participation, and data protection rights. It will be emphasised that the decision of the staff member will not influence or affect their career or work relationships in any way. If the staff member expresses interest in participating, the PI will send an individualised email introducing the staff member to the researcher.

### Data collection

Using a mixed-methods approach, multiple sources of quantitative data will be collected in a sequential manner. Quantitative data collection will take place from service commencement, while qualitative interviewing will take place at two points- approximately 4–6 months after commencement of the service and in the final two months of service provision.

***Quantitative data.*** Quantitative measures will include data that is recorded as a necessary or usual part of palliative care or rehabilitative palliative care service provision during patient assessments in patient charts or electronic patient records. All quantitative measures are summarised in Table 2 under three category headings: demographic data, service-usage data and, health and symptom data. Following the establishment of informed consent from patients, demographic, service use and health and symptom data necessary for the delivery of care will be collected by the service’s occupational therapist and/or physiotherapist. As part of the phased component, survey data on patient satisfaction will be collected on completion of engagement with the rehabilitation service intervention.

***Qualitative data.*** Semi-structured interview schedules for the first set of interviews for each group of participants were developed using a five-step framework of design ^26^. The five steps are as follows: (1) identifying the prerequisites for using semi-structured interviews; (2) retrieving and using previous knowledge; (3) formulating the preliminary semi-structured interview guide; (4) pilot testing the guide; and (5) presenting the complete semi-structured interview guide. Interviews across all three participant cohorts may take place in person or remotely according to the participant’s preference. Only an audio recording will be made regardless of method of interview. Interviews are estimated to last approximately 30–40 minutes and protocols will be piloted with volunteers in St. Francis Hospice to ensure question clarity, in addition to monitoring time demand.

### Data management

Potential participants personal data will be processed under article 6 (1)(e) Public Interest and under article 9 (2)(j) Scientific Research of the General Data Protection Regulation (GDPR) 2016 ^27^, and data will be secured in accordance with the requirements of the Data Protection Act (2015). Data will be anonymised and where applicable, pseudonyms will be used in reports and publications.

***Analytic plan.*** Data analysis will be iterative and will continue over the course of the study.

Descriptive statistics will be generated using R ^28^ to summarize participant characteristics across demographic and service usage domains, where categorical variables will be reported as raw numbers and percentages. Reports of continuous variables will include mean, median, range and standard deviation values. For repeated measures analysis of health and symptom data pre- and post-service engagement, significance of variations will be determined using *x ^2^* tests or Fisher’s Exact Test, when required, for categorical data, Mann-Whitney U tests for ordinal data, and t-tests/ ANOVA for continuous data *. 95%* confidence intervals (CI) will be used, and significance levels will be assessed at the alpha level of *.05.*

Interview audio will be transcribed using Happy Scribe (2021) transcription software and reviewed using original audio-recordings to ensure accuracy, the removal of identifiable information and to engage in data familiarity. For qualitative data, reflexive thematic analysis ^29– 31^ will be used to generate descriptive themes related to the experiences and perspectives of stakeholders on the novel service. Thematic analysis was selected for analysis given its flexibility in application, enabling the collation of differing perspectives and the iterative construction of common themes.

Coding will be conducted both inductively and deductively using the open-source QDA Miner programme *(version Lite),* where inductive coding will entail annotating similarities and intriguing features across the dataset. Deductive coding will comprise the construction of a deductive codebook based on the core foundations of the Adult Palliative Care Services Model of Care ^2^, and contemporary literature on rehabilitation, integration and palliative care ^32– 34^.

Coding will be completed by three researchers forming a coding team with diverse academic backgrounds in health economics, rehabilitative palliative care, and psychology. These researchers will not be involved in the provision of the rehabilitative service. Coding will be completed through individual coding sessions and facilitatory discussions to gauge differences in interpretation and to fully explore the breadth of data collected. As the dataset will be coded progressively, base codes will be continually revised and refined in response to new facets of data. Theme construction will then be facilitated by the design of a coding tree to identify similar concepts generated during the coding process. To ensure regular reflection during the analytic process, a reflexive log will be maintained to track and detail aspects of the coding process to identify potential assumptions underlying their approach ^31^. Qualitative analysis will be reported in line with the COREQ reporting guidelines ^45^.

The construct validity, internal validity, external validity, and reliability of the data will be established through use of the following methods. First, the use of a protocol and exact documentation of each step of the process facilitates traceability ^35^. Second, the use of multiple methods increases validity by providing multiple perspectives on the same phenomenon ^35^. Third, a researcher diary will be used to record thoughts, feelings, and expectations that may at a later stage be used for data analysis ^36^. Fourth, a well-structured database will be used for data management and will serve as the evidentiary source of conclusions ^35^. Fifth, analytical techniques during data analysis such as explanation building and addressing rival explanations will be employed ^37^. Sixth, the multidisciplinary composition of the team will allow the researchers to raise questions throughout the course of the study, particularly regarding the fit between the methods used and the results obtained, and study conclusions ^37^. Finally, thick description will establish transferability ^38^. Convergence of quantitative and qualitative data will be undertaken to describe the service and its implementation from the perspectives of patients, informal carers and clinicians.

### Ethics

The research protocol has been approved by the Mater Misericordiae University Hospital (IRB Ref 1/378/2113) and St Francis Hospice Dublin (Rec. approval 16/1/20).

The case study will be conducted in accordance with the ethical standards of the organisations and with the 1964 Helsinki declaration and its later amendments ^39^. The protocol was designed giving consideration to best practice in palliative and rehabilitative care and ensuring that risks (e.g., participant distress occurring during interview) will be minimised to the greatest possible degree for patients, carers and staff. The autonomy of participants will be respected by providing informed choice. All participants will receive oral and written information prior to the interview, and written informed consent will be obtained prior to interview. Participants will be allotted a minimum time period of 24 hours to consider their interest in participating.Additionally, all participants will be informed that they may withdraw from the study, and/or withdraw their data, at any point without affecting their access to services. All data will be anonymized and will not be identifiable and will be managed securely.

### Public and patient involvement

Due to required timeframes and available resources, public involvement in the development of the case study protocol has been at level one of the Public Participation Spectrum ^40^ developed by the International Association for Public Participation. A virtual meeting was held with members of Voices4Care in March 2021 where the study protocol was presented, and a question-and-answer session held. Voices4Care is an initiative of All Ireland Institute of Hospice and Palliative Care (AIIHPC) – an all-island organisation working to improve palliative and end-of-life care for patients and their families. Voices4Care is a volunteer group with members comprising people living with a life-limiting illness/with palliative care needs, informal carers of adults with palliative care needs, and people from the wider community interested in palliative care.

## Dissemination plans

In keeping with the mission of the research to develop service provision and meet patient needs, knowledge exchange activities will be undertaken to support a dissemination plan that reaches the public, health and social care professionals and policy makers. Dissemination materials will be developed to meet the needs of individual groups and a final project report will be provided to the Sláintecare Programme Implementation Office. Additionally, the case study will be submitted for publication in a peer-reviewed journal. Findings will be presented at national and international meetings. A project webpage has been created and may be accessed at https://palliativerehab.ie/. Information will also be shared via the Rehabilitative Palliative Care Slaintecare Project Twitter feed (@PalliativeRehab).

## Study status

Data collection commenced in February 2020 but was interrupted by the COVID-19 pandemic. The palliative care rehabilitation service was suspended for a three-month period and data collection re-commenced in July 2020. Preliminary data collection is due to finish on December 31 ^st^, 2021,with follow-up interviews with professional staff expected to be completed by May 2022.

## Discussion

Overall, this case study will examine Palliat Rehab by (i) outlining the structures and components of the integrated service, (ii) investigating the perspectives of key stakeholders, and (iii) analysing the chosen outcome measures for suitability in the intervention. Given the projected increases in both palliative care and rehabilitative needs among the Irish population over the next two decades ^1^, this case study is likely to uncover valuable insights into offering an accessible and integrated services to meet these growing demands. Moreover, Palliat Rehab will uphold the aims of Sláintecare reform in the Irish healthcare system, which seeks to achieve a universal single-tier health and social care system where everyone has equitable access to services based on need, and not ability to pay ^53^. Core Sláintecare goals are the achievement of a shift in care from the acute to the community setting to bring care closer to home for service users, and a focus on enablement and well-being. Palliative rehabilitation aligns well with these objectives as it seeks to integrate enablement, self-management, and self-care into the holistic model of palliative care.

Despite being recognised as an essential part of palliative care service provision ^2^, rehabilitation services remain under-developed and under-utilised in Ireland and beyond. New pathways and models of care are required to advance service provision to ensure that patients receive the ‘right care, in the right place, at the right time, by the right people’. The development of ‘‘Palliat Rehab’’ offers opportunity to study an innovative service and consider its potential contribution to the achievement of Sláintecare goals. Investigating the rehabilitation service in-depth and within its environmental context will lead to a better understanding of ‘how’ and ‘why’ things happen. Case study findings will be of value in assessing whether there is evidence that supports the rehabilitation service, and will be used to inform efforts to further develop and tailor the intervention.

### Limitations

Several limitations should be considered. The rehabilitation intervention is being delivered during a period of great change within health services that has been precipitated by the pandemic. This has resulted in a period of suspension of the service but has also impacted on usual ways of working (e.g., the requirement for physical distancing, utilisation of remote forms of communication). Detailed contextual description will be provided to enable others to understand the effect of the pandemic on service provision.

The case study will appraise the suitability of selected outcome measures for use within the rehabilitation service. This is of particular importance given that the clinical usefulness of function and mobility-based outcome measures for individuals receiving specialist palliative care are currently a topic of debate ^41, 42^. Selecting an appropriate outcome measure is a critical step in designing valid and useful clinical trials and outcome studies, as the best design cannot make up for the use of an inappropriate measure. It is possible that the quantitative measures may have limited utility in contributing to knowledge of effectiveness. However, the application of mixed methods methodology will ensure that the strengths of one approach complements the restrictions of another and qualitative data will be used to understand the meaningfulness, or otherwise, of the quantitative outcomes. While the design of the Palliat Rehab service was informed by expert opinion and the evidence base, the staffing of the team was limited by funding availability and the required timelines of the project. Case study findings will provide important data on this issue.

## Conclusion

This case study aims to advance our knowledge of the implementation and delivery of a novel rehabilitative palliative care service in Ireland. By providing an in-depth description of the experiences of patients, carers and health and social care professionals, a better understanding of the ‘how’ and ‘why’ (or ‘why not’) of the service’s perceived effectiveness will be obtained. Findings will be used to develop and tailor the intervention and will inform the development of future interventional studies as part of the journey towards evidence-based service provision.

## Data availability

### Underlying data

No data is associated with this article.

**Extended data**

Open Science Framework: Palliat Rehab Service, https://doi.org/10.17605/OSF.IO/RA93N ^47^

This project contains a description of the novel service in line with the TIDieR checklist, an outline of the core foundations of the Adult Palliative Care Services Model of Care for Ireland and the elements of Palliat Rehab drawing on each foundation, and interview schedules for each sample.

Data are available under the terms of the Creative Commons Zero "No rights reserved" data waiver (CC0 1.0 Public domain dedication).
