# Supplementary material for: Protocol for an exploratory, longitudinal single case study of a novel palliative care rehabilitative service
Source: HRB Open Res. 2022 May 6;4:131. Originally published 2021 Dec 20. [Version 2] doi: 10.12688/hrbopenres.13461.2 (PMC8790707; doi:10.12688/hrbopenres.13461.2)
Supplement: Peer review history

# Peer review: Reviewer response for version 2

**DOI:** 10.21956/hrbopenres.14799.r31989

**Published:** 2022-05-09

**Authors:** JongKyu Kim

**Affiliations:** 1 Department of Physical Medicine and Rehabilitation, Seoul Medical Center, Seoul, South Korea

Thank you for your enthusiasm to this research. I totally agreed with your responses. And I am hoping your next step of the Palliative Rehabilitation program.

Is the study design appropriate for the research question?

Yes

Is the rationale for, and objectives of, the study clearly described?

Partly

Are sufficient details of the methods provided to allow replication by others?

Partly

Are the datasets clearly presented in a useable and accessible format?

Not applicable

Reviewer Expertise:

Rehabilitation for the patients who taken care of pallitive care.

I confirm that I have read this submission and believe that I have an appropriate level of expertise to confirm that it is of an acceptable scientific standard.

# Peer review: Reviewer response for version 1

**DOI:** 10.21956/hrbopenres.14672.r31107

**Published:** 2022-01-25

**Authors:** Jette Thuesen, Mai-Britt Guldin

**Affiliations:** 1 REHPA, The Danish Knowledge Centre for Rehabilitation and Palliative Care, Odense University Hospital, Odense, Denmark; 2 Department of Public Health, Unit for User Perspectives and Community Interventions, University of Southern Denmark, Odense, Denmark; 3 Center for Nutrition and Rehabilitation, Absalon University College, Holbæk, Denmark

Dear Colleagues,

 Thank you for this manuscript. We were excited to read that you had undertaken developing a protocol on a case study of a novel palliative care rehabilitation service. We think this is exactly the type of study that can lift this novel research area. Therefore, we appreciate the subject of your study.

 This is a description of a protocol for a prospective, longitudinal, mixed-methods case-study design that describes a novel early access palliative rehabilitation service. The study will explore the experience of patients, carers, and clinicians by using documentary, survey and interview data.

 While the topic is important and certainly of interest in promoting novel and early access to rehabilitation, the manuscript in its current form suffers from some important limitations which detract from its quality. Revisions are necessary to make the manuscript ready for indexing.

 Below we have commented on some specific points that we find would improve the readability and validity of the study.

**Introduction:**

- Our main point in the introduction is that the references are not clear and the background for a palliative rehabilitation intervention should be clearer. Is reference # 3 a book? 
- It is quoted that there is ‘growing evidence base’ on palliative rehabilitation, but it is not backed with references? A systematic literature search should give you an overview of several palliative rehabilitation intervention studies completed within the last ten years. To mention some, Feldstein and colleagues have published several papers on their interdisciplinary palliative rehabilitation program in 2016-2018.
- We would suggest also visiting the palliative rehabilitation projects from King’s College, London. Finally, we would suggest that the theoretical underpinnings of a palliative rehabilitation service are described better and the reference to The Adult Palliative Care Services Model for Ireland is supplemented with a figure or table that shows its contents. This would lift the understanding of the need for a palliat rehab service further, and make the service model much clearer to the reader and therefore the study protocol more transparent. We would also shorten the introduction regarding the Slaintecare goals in order for it to be interesting to a wider readership, or it needs to be apparent why this is important information to understand this protocol and the reader.
- The aim of the paper is to describe the protocol. However, the authors have chosen to describe the aims of the case study. This gets very confusing. We would suggest describing the aims of this paper clearly – which in our opinion is to present a protocol - and it would be of great help for a clear manuscript that a guidance is used for presenting a protocol. For instance the SPIRIT-statement.org or another guidance. Further, it would also be helpful with a development model or theoretical model to underpin the development and description of the protocol. This should preferably be differentiated from the aims of the case study. Moreover, the development of the palliative rehabilitation intervention might have been framed by a complex intervention development model such as the one described by Bleijenberg et al (2018). This would help you to structure the different background factors for designing the intervention (e.g. problem identification and definition, patient’s needs, theory, evidence etc).  

**Methods:**

- In the description of methods, we think the authors succeed in presenting why the case study design is the most appropriate for the proposed study. Nevertheless, also here the paper needs to be differentiated between this paper/the protocol and the study design which is described for the future study.
- There seems to be some points of confusion regarding the methods (and therefore a guidance on presenting protocols would be helpful): Initially, the study is described as an 18 months period. Later it is described as a 15 month period. The same point about sample size. Initially, it is described as ‘a single, holistic case’. Later it is described as ‘the sample size…depends on the number of participants that is sufficient’ and later again ‘a purposive sample of 16-22 individuals...’. Study setting and recruitment (sample) have repetitions which could be more omitted. These points need to be clearer, more aligned, and maybe even shorter so it is not stating the obvious.
- Additionally, it also seems the section about Sample also describes types of data that will be collected. Consider if these should be described under data collection instead.

**Participation selection and recruitment:**

- Eligibility assessment is described thoroughly and maybe a little lengthy. We would recommend shortening to ensure clarity and readability. Recruitment is also described at length and with other information in between. The recruitment procedure is difficult to get a clear picture of and could even profit from a figure with a flow chart which shows the procedure rather than the lengthy description. This would enhance transparency.
- Be aware that both in Sample and in recruitment, data sources are described. It would make the study more reader friendly to put all description on data sources under data collection.
- Maybe it could also be considered to differentiate between recruitment and study procedure. It seems both types of descriptions are put in the same long section. This makes it harder to understand.
- The section on ‘As discussions involving an individual’s experience with a life-limiting illness…’ is really about ethical considerations and would deserve to be moved to the ethics section of the protocol.
- Finally, we applaud the use of TIDIER to describe the service. However, we miss a whole section on describing the form and content of the intervention. Otherwise, the protocol seems incomplete and too abstract and ultimately uninteresting to a wider readership.

**Data collection:**

- We miss an overview of the case study’s research questions and the methods and materials. It is not clear what is actually being investigated by the methods mentioned.   
- A suggestion could be to add a table that provides an overview of the different phases in the study procedure, the participants and which types of data are collected in the different phases. We had to read these aspects several times to grasp the idea of it.
- According to the health and system data, mentioned in table 2, it is not clear why these specific measures are being applied. We miss some arguments for selecting them as the most relevant measures to evaluate the goals of palliative rehabilitation. We suggest that the authors reflect on what are the relevant goals of palliative rehabilitation – and how should they be measured. 
- Further, all the quantitative measurements scales that are proposed really should be presented with information on whether they are validated and can be compared to a larger data set (as this study only will collect data on very few participants). Measurement points should be presented together with measures.
- It is described, that the bundle of interventions will be investigated rather than specific components. How does that align with the ambition of ‘opening the black box’ as mentioned earlier in the paper.
- Finally, we would suggest to pay attention not only to the patients that are being included in the study but also to the patients choosing not to take part and to the dropouts as well. Previously, palliative rehabilitation intervention studies have documented a remarkable difference between patients who were considered eligible, patients who were included, and patients that completed (Chasen *et al* 2013; Jones *et al *2013).

**Data management:**

- Data analyses of quantitative data should be seen according to the very small N of the study. Hence, there needs to be a reflection of the appropriateness of the statistical analysis plan – preferably with reference to former studies on the subject.

**Public and patient involvement:**

- This section is very hard to understand for a reader outside of Ireland. Authors could consider the international readership of the journal and make this section more widely understandable as there surely are good points to learn from.

**Discussion:**

- This section refers to The Slaintecare Report and Core Slaintecare goals. Unfortunately, as these have not been fully presented in the manuscript, this discussion does not seem readily understandable or even relevant to understand this protocol. Authors need to consider what is relevant information for the reader and for the presentation of the protocol.

**Conclusion:**

- As has been mentioned under introduction and aim, it is also relevant in the conclusion to differentiate between ‘this paper’ and the case study.

Best of luck with your manuscript. 

 Mai-Britt Guldin

 Jette Thuesen

Is the study design appropriate for the research question?

Partly

Is the rationale for, and objectives of, the study clearly described?

Partly

Are sufficient details of the methods provided to allow replication by others?

Partly

Are the datasets clearly presented in a useable and accessible format?

Not applicable

Reviewer Expertise:

Mai-Britt Guldin: Palliative Rehabilitation and Palliative Care Research and Education                                            Jette Thuesen: Palliative Rehabilitation and Rehabilitation Research and Education

We confirm that we have read this submission and believe that we have an appropriate level of expertise to confirm that it is of an acceptable scientific standard, however we have significant reservations, as outlined above.

# Peer review: Reviewer response for version 1

**DOI:** 10.21956/hrbopenres.14672.r31110

**Published:** 2022-01-19

**Authors:** JongKyu Kim

**Affiliations:** 1 Department of Physical Medicine and Rehabilitation, Seoul Medical Center, Seoul, South Korea

Thank you for your great study protocol. It is very interesting and I am looking forward to the result from this study protocol.

 Major recommendations:

1. This study protocol is for the program of 'Palliat Rehab', but showed only a webpage address. If possible, could you explain the summary of the 'Palliat Rehab' program contents?
2. For the data and measures, a previous study for rehabilitation of palliative care patients noticed patients' satisfactions and dissatisfactions (Lee *et al. *(2018 ^1^)). Could you add measurements about patients' and caregivers' satisfaction? 

Is the study design appropriate for the research question?

Yes

Is the rationale for, and objectives of, the study clearly described?

Partly

Are sufficient details of the methods provided to allow replication by others?

Partly

Are the datasets clearly presented in a useable and accessible format?

Not applicable

Reviewer Expertise:

Rehabilitation for the patients who taken care of pallitive care.

I confirm that I have read this submission and believe that I have an appropriate level of expertise to confirm that it is of an acceptable scientific standard, however I have significant reservations, as outlined above.

## References

[1] : Rehabilitation of Advanced Cancer Patients in Palliative Care Unit. *Ann Rehabil Med* .2018;42(1) : 10.5535/arm.2018.42.1.166 166-174 10.5535/arm.2018.42.1.166 29560337PMC5852220

# Response

**Published:** 2022-01-25

**Authors:** Fódhla Ní Chéileachair

**Affiliations:** Mater Misericordiae University Hospital, Dublin, Ireland

Dear Dr. Kim,

 We are delighted you took the time to review the article and welcome your comments!

 In relation to the key components of Palliat Rehab, we would like to direct you to the OSF storage link in the Extended Data section, as a brief description of the service is available as a downloadable document in this file. Indeed, we do stress that differences are likely to be seen between the rehabilitation plans constructed for participants, as the service will be based on individual needs, and provided this service description as a broad overview of the Palliat Rehab programme.  We also agree that an evaluation of patients' and caregivers' satisfaction would be a welcome addition. Unfortunately, the programme is currently underway, however we are keen to gather qualitative data on the experiences of patients and their caregivers using semi-structured interviews.

 Many thanks for your contribution and your review. We look forward to disseminating findings of the current programme soon!

 With best wishes,

 Fódhla Ní Chéileachair

# Peer review: Reviewer response for version 1

**DOI:** 10.21956/hrbopenres.14672.r31108

**Published:** 2022-01-12

**Authors:** Lubayna Fawcett

**Affiliations:** 1 Physical Therapy Department, Mayo Clinic, Scottsdale, AZ, USA; 2 Arizona State University, Tempe, AZ, USA

This article will contribute to the much needed 'way forward' in palliative rehabilitation. The study settings and sampling as well as the outcome measures tools can all be easily implemented/replicated even in low income countries. 

 In addition, this article underscores the essence of comprehensive palliative care, in which  palliative rehabilitation is the critical and missing component by which to improve the quality of care for people with serious illnesses.

Is the study design appropriate for the research question?

Yes

Is the rationale for, and objectives of, the study clearly described?

Yes

Are sufficient details of the methods provided to allow replication by others?

Yes

Are the datasets clearly presented in a useable and accessible format?

Not applicable

Reviewer Expertise:

Palliative Rehabilitation/Cancer Care/Women's Health.

I confirm that I have read this submission and believe that I have an appropriate level of expertise to confirm that it is of an acceptable scientific standard.

# Response

**Published:** 2022-01-25

**Authors:** Fódhla Ní Chéileachair

**Affiliations:** Mater Misericordiae University Hospital, Dublin, Ireland

Dear Dr. Fawcett,

 We're delighted with your comments on the protocol. Many thanks for taking the time to review this publication.

 With best wishes,

 Fódhla Ní Chéileachair
